# Immunoenhancement Effects of Glycosaminoglycan from *Apostichopus japonicus*: In Vitro and In Cyclophosphamide-Induced Immunosuppressed Mice Studies

**DOI:** 10.3390/md15110347

**Published:** 2017-11-07

**Authors:** Han Wang, Shuang Yang, Yuanhong Wang, Tingfu Jiang, Shuai Li, Zhihua Lv

**Affiliations:** 1 School of Medicine and Pharmacy, Ocean University of China, Qingdao 266003, Shandong, China; wanghan0812@sina.com (H.W.); yangshuang@ouc.edu.cn (S.Y.); yhwang@ouc.edu.cn (Y.W.); jiangtingfu@ouc.edu.cn (T.J.); lishuai890126@126.com (S.L.); 2 Laboratory for Marine Drugs and Bioproducts of Qingdao National Laboratory for Marine Science and Technology, Qingdao 266003, Shandong, China; 3 Key Laboratory of Marine Drugs, Ministry of Education of China, Qingdao 266003, Shandong, China

**Keywords:** sea cucumber, glycosaminoglycan, cyclophosphamide, immunomodulation

## Abstract

In this study, the immunomodulatory activities of *Apostichopus japonicus* glycosaminoglycan (AHG) on the nature killer (NK) cells, cytotoxic T lymphocytes (CTLs) and cyclophosphamide (CY)-treated mice were investigated. After stimulation with multiple concentrations of AHG (0–100 μg/mL), NK cells and CTLs displayed outperformance against YAC-1 and B16 cells, respectively. Furthermore, the mitogen-induced splenic lymphocyte proliferation in CY-induced immunosuppressed mice was significantly promoted by AHG. In addition, the administration of AHG dramatically increased the splenocytes Ca^2+^ concentration and the delayed-type hypersensitivity (DTH) reaction in a dose-dependent manner. Besides, AHG could strongly increase the total antioxidant capacity (T-AOC), the activities of superoxidase dismutase (SOD), catalase (CAT) as well as glutathione peroxidase (GSH-PX), and could decrease the malondialdehyde (MDA) level in the heart, kidney and liver. These findings indicated that AHG played an important role in the immune enhancement and protection against CY-induced immunosuppression and oxidative damage. Our findings provide experimental evidence for further research and possible immunostimulatory applications of AHG in clinical practice.

## 1. Introduction

Immunomodulatory agents used as complementary or alternative medicines have become popular for treating different immune disorders. Especially, co-administration of immunomodulatory agents and anti-tumor drugs is used to reduce the harmful side effect of chemotherapy [1]. Numerous natural substances extracted from plants or animals were found to be beneficial to ameliorate disease symptoms by stimulating both innate and adaptive immunity. Among these, the bioactive polysaccharides isolated from natural source have recently been studied as a new immunopotentiator source for their profound effect on the immune system with relative nontoxicity and no significant side effects [2]. They exert a variety of immune regulatory functions including the activation of immune-related cells, promotion of cytokine or chemokine secretion as well as activation of complement system [3]. Until now, various polysaccharides have been applied as immunomodulators in clinic, such as lentinan (LNT) [4], krestin (PSK) [5,6], *Astragalus* polysaccharides (APS) [7] and *Panax ginseng* polysaccharides (GPS) [8].

Many polysaccharides derived from marine organisms have novel structural characteristics and possess excellent biological functionalities. Holothurian glycosaminoglycan (HG), which is the dominated component of sea cucumber, exhibited multiple bioactivities, such as anticoagulation [9,10], immune regulation [11,12], anti-cancer [13,14] and antiviral activities [15]. The sea cucumber *Apostichopus* (*Stichopus*) *japonicus* is the most popular source of fucosylated chondroitin sulfates (FCS). The different environment and extraction process may influence the fine structural characterization of glycosaminoglycans from the sea cucumbers *Apostichopus* (*Stichopus*) *japonicas* [16,17,18]. In our previous study, we isolated a novel glycosaminoglycan from *Apostichopus japonicus* (AHG) (Mw 98.07 kDa), the structure of which contained a chondroitin sulfate-like backbone together with large quantity of one fucopyranosyl residue attaching to the 3-O position of β-d-glucuronic (GlcA) and 4-O and/or 6-O positions of *N*-acetyl-β-d-galactosamine (GalNAc). The molar ratio of GlcUA, GalNAc, Fucose (Fuc) and sulfate of AHG was 0.97:1.00:1.13:3.85 [18]. In addition, it was revealed that AHG has not only immunoregulation capability on both innate and adaptive immune in vitro, but also protective effects toward the hematopoietic function of bone marrow and immune organs in myelosuppressed mice [19,20,21]. However, the protective effects of AHG on immunological effector cells and organs against immunosuppression and oxidative damage are poorly understood.

In present study, we investigated the effect of AHG on anti-tumor activity of nature killer (NK) cells and cytotoxic T lymphocytes (CTLs) in vitro. Furthermore, the immunomodulatory effects of AHG on spleen lymphocyte proliferation, cytokines secretion, intracellular free Ca^2+^ concentration (second messengers to activate lymphocytes), delayed-type hypersensitivity (DTH) reaction, as well as antioxidant activity in cyclophosphamide (CY)-induced immunosuppressed mice were also systematically elucidated in vivo.

## 2. Results

### 2.1. Cytotoxic Effect of AHG on Splenocytes

**Figure 1:**
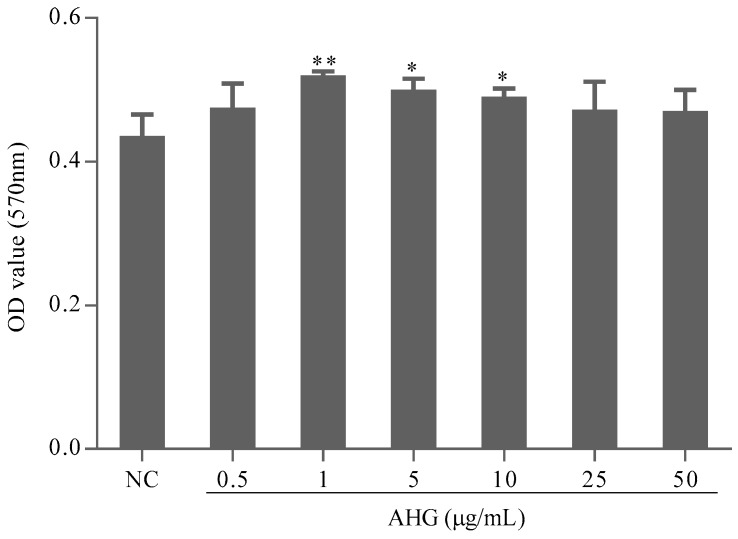
Effect of glycosaminoglycans from *Apostichopus japonicus* (AHG) on splenocytes viability and proliferation in vitro (*n* = 8). Compared with the NC group * *p* < 0.05, ** *p* < 0.01.

The absorbance at 570 nm (A570) measured by 3-(4,5-dimethylthiazol-2-yl)-2,5-diphenyltetrazolium bromide (MTT) assay can reflect the splenocytes viability. The A570 values of AHG groups did not decrease compared with those of normal control (NC) group (Figure 1). It was suggested that AHG at 0.5–50 μg/mL had no toxic effect on mouse splenocytes. On the contrary, AHG at 1–10 μg/mL significantly increased the amount of splenocytes, which indicated that treatment of AHG at low concentration could promote splenocytes proliferation responses.

### 2.2. Effect of AHG on Splenic NK Cells Activity

**Figure 2:**
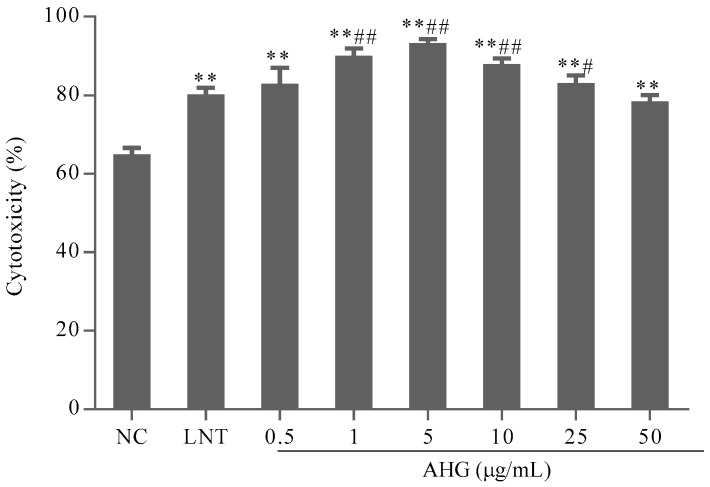
Effect of AHG on splenic nature killer (NK) cells cytotoxicity against Yac-1 lymphoma cells in vitro (*n* = 8). Compared with the NC group ** *p* < 0.01; compared with the lentinan (LNT) group ^#^ *p* < 0.05, ^##^ *p* < 0.01.

To evaluate the effect of AHG on tumor cell elimination mediated by NK cells, the cytotoxicity of splenocytes against NK cells-sensitive YAC-1 lymphoma cells was investigated. As shown in Figure 2, compared with the NC group, the treatment with AHG (0.5–50 μg/mL) enhanced NK cells cytotoxicity significantly (*p* < 0.01). The maximum effective concentration was 5 μg/mL. The cytotoxicity of NK cells in AHG groups with the concentration ranged from 1 to 25 μg/mL were higher than that in LNT (40 μg/mL) group (*p* < 0.05). These results indicated that AHG strengthened the activity of NK cell against tumor cells.

### 2.3. Effect of AHG on Splenic CTLs Activity

The phenotypic profile of a representative population of bone marrow dendritic cell (DCs) was determined using flow cytometer. After DCs were cultured in the presence of GM-CSF and IL-4 for seven days, they differentiated into mature DCs that expressed high levels of CD80 antigen, from 2.17 to 83.81%.

**Figure 3:**
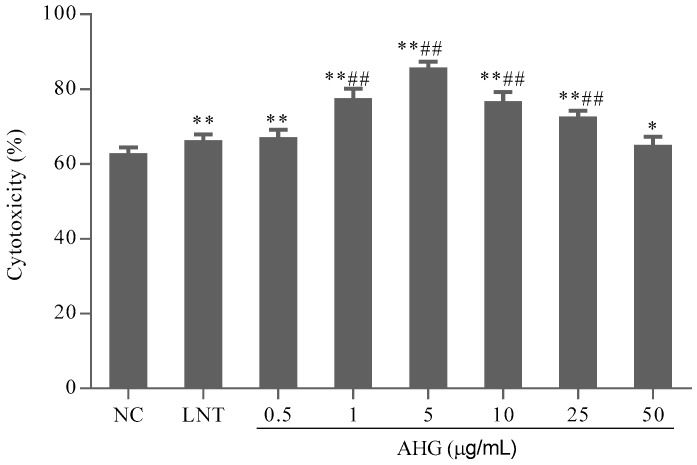
Effect of AHG on dendritic cell (DC)-mediated- cytotoxic T lymphocytes (CTL) activity in vitro (*n* = 8). Compared with the NC group * *p* < 0.05, ** *p* < 0.01; compared with the LNT group ^##^ *p* < 0.01.

B16 melanoma cells were used as the target cells to evaluate the specific cytotoxicity of activated-CTLs. The results shown in Figure 3 indicated that the cytotoxicity of CTLs in AHG groups ranged from 0.5 to 25 μg/mL, significantly higher than that in NC group. AHG at 5 μg/mL possessed the highest cytotoxicity (*p* < 0.01). Thus, AHG could enhance the cytotoxic of CTL stimulated by DCs on melanoma cells in different degree.

### 2.4. Effect of AHG on Mitogen-Induced Splenic Lymphocyte Proliferation in CY-Treated Mice

**Figure 4:**
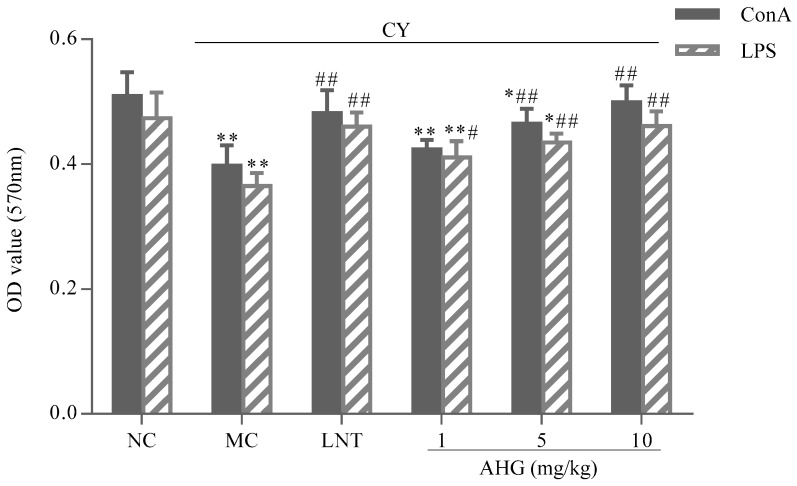
Effect of AHG on mitogen-induced splenic lymphocytes proliferation in CY-treated mice (*n* = 8). Compared with the NC group * *p* < 0.05, ** *p* < 0.01; compared with the LNT group ^#^ *p* < 0.05, ^##^ *p* < 0.01.

The effects of AHG on mitogen-stimulated murine spleen lymphocyte proliferation were presented in Figure 4. Apparently, the proliferative responses of lymphocytes induced by both Concanavalin A (Con A) and Lipopolysaccharide (LPS) were reduced remarkably in CY-treated mice (*p* < 0.01) compared with the NC group. Combining with Con A or LPS, AHG could enhance the proliferation of splenocytes in a dose-dependent manner. The 5 and 10 mg/kg AHG combined with Con A significantly promoted and strengthened the proliferation of the splenocytes compared with that of MC group (*p* < 0.01). Similar result was also found in the stimulation of AHG combined with LPS on lymphocyte proliferation, while, in synergistic stimulation with LPS, AHG provided obvious increase of the splenocyte proliferation at 1, 5 and 10 mg/kg compared to the MC group (*p* < 0.05). Especially at the dose of 10 mg/kg, the A570 values were nearly equal to that of normal mice. These results indicated that co-mitogenic activities of AHG, with either Con A or LPS, were dose-dependent.

### 2.5. Effects of AHG on Cytokines Secretion of Splenocytes in CY-Treated Mice

**Figure 5:**
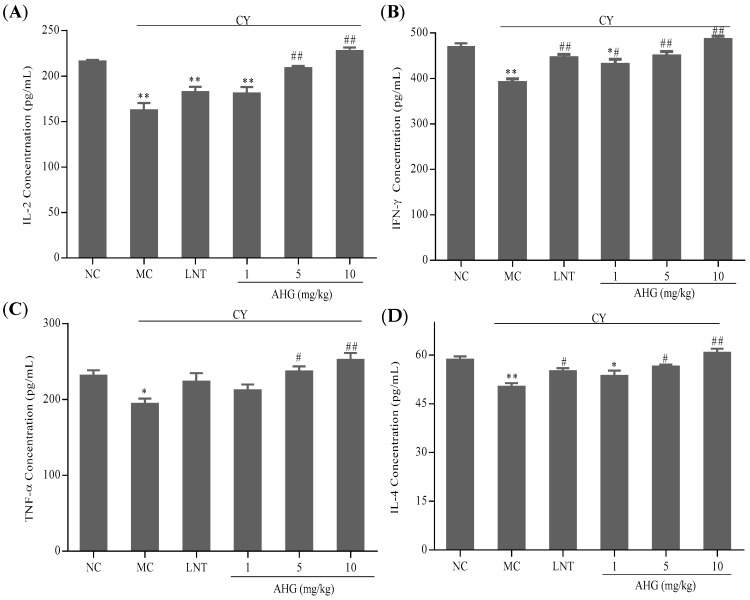
Effects of AHG on cytokines secretion of splenocytes from CY-treated mice (*n* = 8): (**A**) IL-2; (**B**) IFN-γ; (**C**) TNF-α; and (**D**) IL-4. Compared with the NC group * *p* < 0.05, ** *p* < 0.01; compared with the LNT group ^#^ *p* < 0.05, ^##^ *p* < 0.01.

The results in Figure 5 showed that the cytokines including interleukin-2 (IL-2), interferon-γ (IFN-γ), tumor necrosis factor-α (TNF-α) and interleukin-4 (IL-4) secreted by splenocytes in CY-treated mice were lower than those in normal animal (*p* < 0.01). However, there was a dose-dependent increase of cytokines production after AHG administration in CY-treated mice. More interestingly, the 10 mg/kg AHG provided obvious promotion and strengthening of cytokines (IL-2, IFN-γ, TNF-α and IL-4) secretion (*p* < 0.01), and led to slight increases versus values seen in the normal control mice.

### 2.6. Effect of AHG on Intracellular Free Ca^2+^ Concentration of Splenocytes in CY-Treated Mice

**Figure 6:**
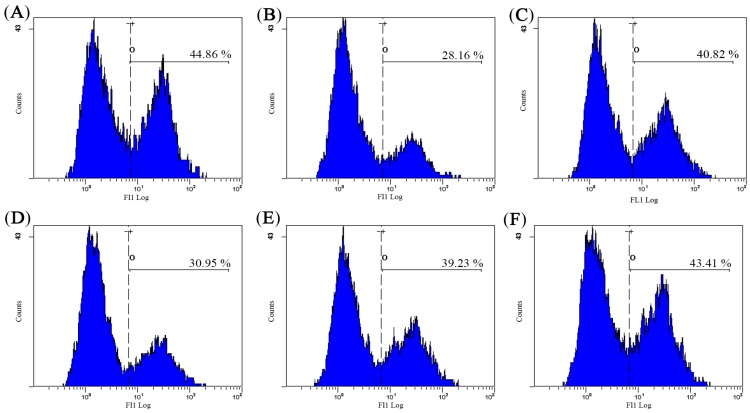
Effect of AHG on intracellular free Ca^2+^ concentration in spleen lymphocytes of CY-induced mice (*n* = 8): (**A**) NC group; (**B**) MC group; (**C**) LNT group; (**D**) AHG group at 1 mg/kg; (**E**) AHG group at 5 mg/kg; and (**F**) AHG group at 10 mg/kg.

As shown in Figure 6, the intracellular free Ca^2+^ concentration was lowest in the model control (MC) group. The treatment of AHG resulted in dose-dependent increase in the amount of Ca^2+^ when compared with MC group. Particularly, the level of Ca^2+^ in AHG group at 10 mg/kg nearly reached the level in NC group. These results showed that AHG was capable of reversing the decrease of Ca^2+^ concentration in CY-treated mice to the normal level.

### 2.7. Effect of AHG on DTH Reaction in CY-Treated Mice

**Figure 7:**
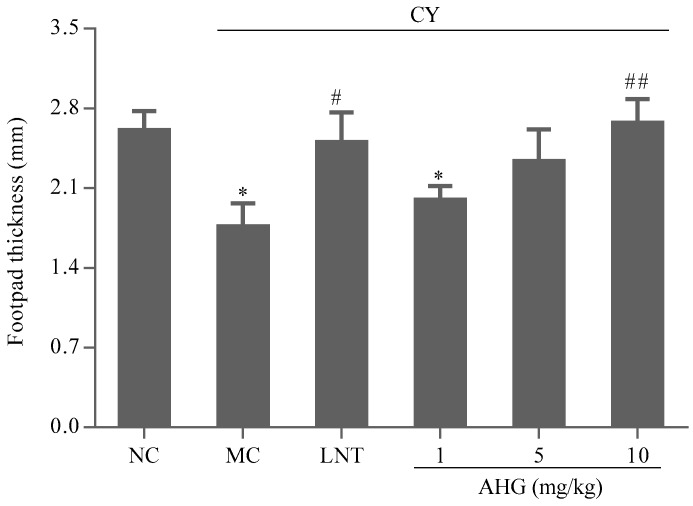
Effect of AHG on DTH reaction in CY-treated mice (*n* = 8). Compared with the NC group * *p* < 0.05; compared with the LNT group ^#^ *p* < 0.05, ^##^ *p* < 0.01.

The effect of the AHG treatments on the DTH reaction to sheep red blood cells (SRBC) in mice was displayed in Figure 7. The footpad thickness of mice in MC group was markedly lower than that in NC group (*p* < 0.05). AHG treatment exhibited an enhancement on footpad edema volume in CY-treated mice. Significant change was observed at the highest dose of AHG (10 mg/kg, *p* < 0.01). Thus, it was suggested that AHG strengthened the T-cell-mediated immune response in mice.

### 2.8. Antioxidant Activity of AHG in CY-Treated Mice

CY could induce free radical production, followed by cytotoxicity and oxidative stress. As shown in Table 1, Table 2 and Table 3, CY significantly reduced the T-AOC, SOD, CAT and GSH-Px activities in hearts, livers and kidneys. In comparison with MC group, AHG administration could enhance the T-AOC, SOD, CAT and GSH-Px in different tissues dose-dependently. Particularly, the level of T-AOC, SOD, CAT and GSH-Px were almost recovered to normal level when treated with AHG at the concentration of 10 mg/kg (*p* < 0.01). Meanwhile, Table 1, Table 2 and Table 3 showed the significant increases in MDA levels in the hearts, livers and kidneys in mice of CY-treated group (*p* < 0.01), while AHG treatment at 1, 5, and 10 mg/kg could cause a dose-dependent decrease in the accumulation of tissue MDA. In addition, when the dose of AHG reached to 10 mg/kg, the contents of MDA were recovered to the normal levels. The results suggested that AHG can enhance anti-oxidative activities in liver, kidney and heart.

## 3. Discussion

In the present study, we for the first time reported the immunostimulatory activities of a novel glycosaminoglycan fraction isolated from *Apostichopus japonicus* on NK cells, CTLs in vitro and immunosuppressed mice caused by cyclophosphamide treatment.

NK cells and CTLs are two major populations of cytotoxic lymphocytes, and play important roles in the control of tumor growth and metastasis [22,23]. In this work, IL-2 (also termed T-cell growth factor) was used to enhance the generation and cytotoxic activity of NK and CTL cells in vitro [24,25]. NK cells can efficiently kill cells without previous sensitization. Obviously, treatment with AHG dose-dependently accelerated the cytotoxicity of NK cells to Yac-1 lymphoma cells in this study. DCs are the most potent professional antigen presenting cells that can induce antigen-specific CTL immune responses and regular adaptive immune response, as well as participate in differentiation of T cells subset and inherent immunity response [26,27]. Tumor lysate-pulsed DCs presented tumor antigens to T cells by both major histocompatibility complex (MHC) class I- and class II-pathways, and then provide the potential to induce efficient antitumor immune responses [28]. Flow cytometry analysis showed that these DCs, which expressed high levels of CD80 antigen, had typical mature phenotypic markers. Functionally, these cells gained the capacity to stimulate allogeneic T cells. The results indicated that primed T cells in vitro with B16 melanoma cells lysate-pulsed DCs were able to induce specific CTL against B16 tumor cells. Especially, after stimulated with various concentration of AHG, the cytotoxicity of specific-CTL were much stronger than that in NC group. Taken together, it was demonstrated that AHG can significantly promote NK cells and specific-CTL antineoplastic activity.

We further evaluated the immunomodulatory effects of AHG in a CY-induced immunosuppression murine model. CY, an alkylating agent, is an important chemotherapeutic drug in malignant tumor treatment. However, CY intake can injure DNA of normal cells and cause myelosuppression, immunosuppression and oxidative stress on various tissues, which sometimes are life-threatening [1,29]. In the present study, we used CY as an immunosuppressive agent to establish a model of weakened immunity. As expected, CY resulted in immunodeficiency, as evidence by significantly reducing splenocyte proliferation, cytokine secretion, intracellular free Ca^2+^ concentration, and inhibiting SRBC-induced DTH reaction as well as damaging antioxidant system. These remarkable differences in various immune parameters between the CY-treated group and the NC group indicated that the immunosuppression model was applicable for in vivo experiments.

Splenocyte proliferation is a crucial event reflecting both cellular and humoral immune response because of its considerable sensitivity [30]. Lymphocytes stimulated by Con A in vitro may be used to evaluate T lymphocyte activity associated with cellular immunity, while those stimulated by LPS may be used to evaluate B lymphocyte activity, which participate in the humoral immunity [31]. In this study, CY-induced suppression of Con A-induced T-lymphocyte proliferation and LPS-induced B-lymphocyte proliferation were recovered by AHG. Proliferation assay results suggested that AHG can significantly increase the activation potential of T and B cell proliferation and enhance the immune response in immunosuppression mice.

The activated splenic lymphocytes play an important role in the innate and adaptive immuneresponses by producing cytokines [29]. Cytokines are important in cell–cell communication in the immune system and play key roles in improving the body’s defense mechanism. The T helper (Th) cells are divided into Th1 and Th2 according to the function and their difference in secretion of cytokines. Th1 cells secrete IL-2, IL-12, IFN-γ, and TNF-α, participating in cell-mediated immune responses, while Th2 cells secrete IL-4, IL-5, IL-6, and IL-10, which promote humoral or allergic responses. IL-2, originally described as T-cell growth factor, stimulates the proliferation and differentiation of T cells and increases the IFN-γ secretion of NK cells [32,33]. IFN-γ is a pro-inflammatory cytokine endowed with potential immunomodulatory effects on Th1 cells differentiation and macrophage activation to acquire microbicidal and antiviral effector functions [34,35]. IL-4 is a critical participant in allergic inflammation. It induces the differentiation of Th cells into Th2 cells and the growth of B cells [36]. TNF-α, discovered by its antitumor activity, acts as a host defence factor in immunologic and inflammatory responses [37]. The present results showed that cytokines (IL-2, TNF-α, IL-4 and IFN-γ) expression in the AHG-treated group was much higher than that in CY-treated group and high-dose AHG-treat (10 mg/kg) could secrete more cytokines (IL-2, TNF-α, IL-4 and IFN-γ) compared with normal control, suggesting that AHG can reversed the splenocytes function reduced by CY. In addition, AHG can active Th1 and Th2 cell at the same time.

Calcium ions, known as the most widely used intracellular secondary messengers, play an essential role in lymphocyte function, which participate in proliferation, differentiation and gene transcription of lymphocyte [38,39]. The elevation in the concentration of Ca^2+^ in the cytosol triggers the activation and proliferation of lymphocytes, especially promotes the transcription factors translocating from cytoplasm to the nucleus and binds to the promoter. The final consequence is initiating the transcription of specific cytokine genes [40]. It has been mentioned that PSG-1, the polysaccharide obtained from *Ganoderma atrum*, may activate spleen lymphocytes via Ca^2+^/CaN/NFAT/IL-2 signaling pathway, similar to the polysaccharide fraction of *Panax ginseng* [41,42]. In our study, the concentrations of Ca^2+^ were noticeably increased after AHG administration. Particularly, Ca^2+^ concentration in the AHG group at 100 mg/kg reached the level of that in NC group. These results showed that AHG was capable of recovering the decrease of the concentration of Ca^2+^ in CY-treated mice to the normal level, which may be the root of the enhancement of splenocyte cytokines secretion.

Delayed-type hypersensitivity (DTH), as the fourth type of hypersensitivity reaction, is an important type of cell-mediated pathologic response, and plays a pivotal role in evaluating T cell-mediated immune responses [43,44]. In this research, SRBC were used to induce footpad DTH reaction. We found that AHG potentiated the SRBC-induced DTH reaction in footpads of CY-treated mice and counteracted the inhibitory effect of CY on the DTH reaction. The foot volume was increased after AHG treatment, suggesting the cell-mediated immune in CY-treated mice was enhanced.

Accumulating evidence strongly suggests that many polysaccharides have immunomodulatory activity usually accompanying with antioxidant activity, such as polysaccharides from *Dietary litchi* pulp [45], *Cordyceps militaris* [46], and *Polygoni Multiflori* Radix [47]. The CY-induced overproduction of oxidant compounds associates with the inflammatory response, and can lead to reduced function of virtually all immune cells [48]. Aberrant production or regulation of reactive oxygen species (ROS) cause tissue damage and loss of function in a number of tissues and organs [49]. The lipid peroxidation decreases membrane fluidity, which adversely affects immune responses. Therefore, the relevance of antioxidants is particularly critical for the functionality of immune system [50]. The antioxidant enzymes such as SOD, CAT and GSH-Px in tissues can convert active oxygen molecules into non-toxic compounds to protect against oxidative stress and tissue damage. T-AOC reflects or represents the capacity of the non-enzymatic antioxidant defense system. MDA, involved in forming lipid radicals and oxygen uptake, is a marker for endogenous lipid peroxidation [45]. In our present study, CY treatment resulted in the suppression of T-AOC, SOD, CAT and GSH-Px in heart, liver and kidney as well as an increase in the MDA level. However, the treatment of AHG (1, 5 and 10 mg/kg) significantly increased the levels of T-AOC, SOD, CAT and GSH-Px as well as decreased the MDA levels in tested tissues. These findings showed that AHG can be effective in scavenging various types of oxygen free radicals and their products, indicating that AHG was able to protect against oxidative stress induced by CY in vivo. Further investigation is necessary to verify the precise repair mechanism of AHG and the interaction with other medicines.

## 4. Materials and Methods

### 4.1. Chemicals and Materials

AHG was prepared as the protocols previously described [16]. RMPI 1640 was purchased from Hyclone (Thermo Fisher, Shanghai, China). Fetal bovine serum was purchased from Gibco (Thermo Fisher, Shanghai, China). Con A, LPS, penicillin and streptomycin were purchased from Sigma-Aldrich (St. Louis, MO, USA). CY and 3-(4,5-dimethylthiazol-2-yl)-2,5-diphenyltetrazolium bromide (MTT) was obtained from Solabio (Beijing, China). Anti-mouse FITC-labeled CD 80 monoclonal antibodies was purchased from eBioscience (San Diego, CA, USA). Mouse IL-2, IL-4, TNF-α, IFN-γ Enzyme-Linked Immunosorbent Assay (ELISA) kit was obtained from Dakewe (Beijing, China). Fluo-3/AM fluorescent probe and BCA protein assay kit was purchased from Beyotime (Shanghai, China). Dimethyl sulfoxide (DMSO) was purchased from Sangong Biotech (Shanghai, China). GSH-PX, SOD, CAT, T-AOC and MDA kits were purchased from Nanjing Jiancheng Bioengineering Institute (Nanjing, China). Injectable LNT, used as positive control, was purchased from Nanjing Easeheal Pharmaceutical Co., Ltd. (Nanjing, China).

### 4.2. Animals

Male Kunming mice weighing 25–30 g were purchased from Qingdao Institute for Drug Control (Qingdao, China, SCXK2009007), and maintained under controlled conditions (temperature: 25 ± 2 °C, humidity: 50 ± 5%, 12 h dark-light cycle). The animals were acclimated for 7 days with free access to standard diets and sterile water. All of the animal experiments adhered to strict compliance according to Animal Ethics Committee of School of Medicine and Pharmacy, Ocean University of China for the use and care of animals.

### 4.3. Immunomodulatory Activity of AHG In Vitro

#### 4.3.1. Cell Preparation and Culture

The extirpated spleen was removed in germ-free condition, and gently grinded in aseptic phosphate-buffered saline (PBS) through a stainless steel meshes. The splenocyte suspensions were resuspended in erythrocyte lysis buffer for 5 min to remove the red blood cells. After centrifuged at 300 *g* for 5 min, cell numbers and viability (over 95%) were assessed microscopically using trypan blue dye exclusion technique.

B16 melanoma cells and Yac-1 lymphoma cells were obtained from institute of cell biology, Chinese academy sciences (Shanghai, China). Cells were cultured in RPMI 1640 medium supplemented with penicillin/streptomycin (100 IU/mL and 100 μg/mL, respectively) and 10% heat-inactivated FBS in an atmosphere of 5% CO_2_ and 90% relative humidity at 37 °C.

#### 4.3.2. Cytotoxic Effect of AHG on Splenocyte

The splenocytes (5 × 10^6^/mL) were stimulated with serial concentrations of AHG (0.5–50 μg/mL) for 24 h at 37 °C. The cells treated with the medium alone were used as normal control. The cytotoxic effect of AHG on splenocyte cells was measured by MTT assay. The experiment was repeated three times.

#### 4.3.3. Cytotoxicity Assays of NK Cell Activity of Splenocytes

The splenocytes were activated for 24 h in the presence of IL-2. For cytotoxicity assays, cells (effector cells, 5 × 10^6^/mL) were further co-cultured with YAC-1 (target cells, 5 × 10^5^/mL) in the 96-well plates at 37 °C in 5% CO_2_. Each well was added various concentrations of AHG, or LNT (40 μg/mL, positive control) and medium alone, respectively. After 20 h, the activity of NK cell was determined by MTT assay and calculated by the following formula: NK cell activity (%) = [OD^T^ − (OD^S^ − OD^E^)]/OD^T^ × 100%, where OD^T^ is optical density value of target cells control, OD^S^ is optical density value of test samples and OD^E^ is optical density value of control effector cells.

#### 4.3.4. Assay of CTL Activity of Splenocytes

Primary bone marrow-derived DCs were flushed from the femurs and tibiae of mice in sterile conditions, and incubated in RPMI 1640 medium at 37 °C in 5% CO_2_. On Day 3, non-adherent cells were discarded. The cells were further cultured for 4 days in fresh medium containing GM-CSF (10 ng/mL) and IL-4 (10 ng/mL). On Day 7, DCs were incubated in the presence of freeze-thawed B16 tumor lysates. B16 melanoma cells were lysed by rapid freezing (liquid nitrogen) and thawing at 37 °C in physiological saline three times. After 24 h, LPS (1 μg/mL) was added into the culture for DC-maturation for another three days. The mature B16 melanoma cells lysate-pulsed DCs were harvested, which was determined using- flow cytometer (Beckman Coulter, Brea, CA, USA) after incubating with CD80-FITC.

To examine CTL activities, splenocytes (1.0 × 10^6^/mL) were harvested and then primed in vitro for 1 h in the presence of IL-2 (10 ng/mL). Those cells were incubated with mature DCs (1 × 10^5^/mL) at a ratio of 10:1 in 96-well plates for 3 days to obtain specific-CTL cells. The specific CTLs (effector cells, 5 × 10^4^/mL) were collected and mixed with B16 cells (target cells, 5 × 10^3^/mL) into 96-well plates to give effector/target cells a ratio of 10:1. The cells were treated with various concentrations of AHG for 44 h. The CTL cell cytotoxicity was measured by MTT assay. The activity of CTLs was calculated by the following formula: CTL cell activity (%) = (OD^T^ − (OD^S^ − OD^E^))/OD^T^ × 100%, where OD^T^ is optical density value of target cells control, OD^S^ is optical density value of test samples and OD^E^ is optical density value of control effector cells.

#### 4.3.5. MTT Assay

The cells were seeded in 96-well plates and cultured as mentioned above. Then, 20 μL MTT (5 mg/mL) was added to each well. The plates were incubated for 4 h. DMSO solution (150 μL) was added to resolve the colored material, and the absorbance of each well was measured at 570 nm on a microplate reader (BioRad 680, Hercules, CA, USA).

### 4.4. Immunomodulatory Activity of AHG In Vivo

#### 4.4.1. Animal Experiments

The mice were randomly divided into six groups with 8 animals for each. One group was selected as NC group, which was treated with physiological saline, while the other five groups of mice were subjected to immunosuppression by administration of CY (100 mg/kg) intraperitoneally for 3 days. Then mice in AHG groups were intraperitoneally injected AHG at the dose of 1, 5 or 10 mg/kg body weight, respectively, once a day for the next 7 consecutive days, while mice in positive control group were treated with 1 mg/kg body weight LNT. Other mice and model control (MC) group were only given physiological saline at the same intervals. The animals were sacrificed by decapitation at 24 h after the last administration and heart, liver, kidney and spleen tissues were excised rapidly for further analysis.

#### 4.4.2. Splenic Lymphocyte Proliferation Assay

The splenocytes prepared as described above were adjusted to a final density of 5 × 10^6^/mL, and stimulated with or without Con A (5 μg/mL) and LPS (10 μg/mL), respectively. The cells were incubated in 96 well plates at 37 °C in an incubator with 5% CO_2_ for 48 h. Lymphocyte proliferation was assessed by the absorbance at 570 nm through following MTT assay.

#### 4.4.3. Assay of Cytokine Levels

The cytokines (IL-2, TNF-α, IFN-γ and IL-4) of the collected supernatants from splenocytes culture medium was determined using ELISA kits according to the manual of the manufacturer. The concentrations of IL-2, TNF-α, IFN-γ and IL-4 were elucidated by the absorbance values at 450 nm.

#### 4.4.4. Measurement of Intracellular Ca^2+^

Splenocytes (1 × 10^6^/mL) were seeded in the 24-well plate, and incubated with the fluorescent calcium prober Fluo-3/AM at 37 °C for 45 min. Post incubation, the cells obtained by centrifugation were washed twice with PBS. Fluorescent signals were then detected by flow cytometry.

#### 4.4.5. Assay of DTH Reaction (Footpad Reaction Test)

Before three days of finishing administration, all the mice were immunized intraperitoneally with 0.2 mL of 5% (*v*/*v*) SRBC. On 9th day, after measuring the left footpad thickness, the mice were again challenged with 20 μL of 20% (*v*/*v*) SRBC in another paw. The increase footpad thickness was considered as foot swelling (in mm) after 24 h.

#### 4.4.6. Biochemical Assays

The homogenate of liver, heart or kidney was prepared in 0.1 g/mL wet weight of ice-cold physiological saline. The samples were centrifuged at 2000 *g* at 4 °C for 10 min, and the supernatants were collected for the measurement of protein, T-AOC, MAD, CAT, SOD and GSH-Px. These antioxidant parameters were determined by commercially available assay kits in accordance with the manufacturer protocols.

### 4.5. Statistical Analysis

All results were expressed as means ± standard deviation (SD). Statistical significance of different group was evaluated by the use of one-way analysis of variance (ANOVA) using IBM SPSS statistics version 19.0 (IBM SPSS Inc., Chicago, IL, USA). P values less than 0.05 were considered statistically significant.

## 5. Conclusions

The current study demonstrated that holothurian glycosaminoglycan (AHG) could successfully improve immunoregulation activity in vitro, and possesses the capacity to restore CY induced immunosuppression in vivo. Based on these findings and the data accumulated, it was suggested that AHG has huge potential to be an effective immunomodulatory agent and adjuvant in clinical treatment of all cancer patients. Further investigation should be conducted to understand the overall intracellular process associated with the AHG-induced immune response for the development of a novel marine medicine.

## Figures and Tables

**Table 1:** Effects of AHG on antioxidant activity in livers from CY-treated mice (*n* = 8).

Group	Dose (mg/kg)	Malondialdehyde (MDA, nmol/mg pro)	Superoxidase Dismutase (SOD, U/mg pro)	Total Antioxidant Capacity (T-AOC, U/mg pro)	Glutathione Peroxidase (GSH-PX, U/mg pro)	Catalase (CAT, U/mg pro)
NC		1.40 ± 0.18	383.26 ± 15.34	4.30 ± 0.23	213.22 ± 15.56	51.07 ± 3.78
MC		3.09 ± 0.18 **	280.17 ± 33.59 **	2.46 ± 0.22 **	147.68 ± 19.88 **	23.79 ± 3.91 **
LNT	1	2.06 ± 0.14 **^##^	368.63 ± 27.40 ^##^	4.11 ± 0.14 ^##^	211.75 ± 22.31 ^##^	34.72 ± 3.13 **^##^
AHG	1	2.81 ± 0.17 **^#^	283.71 ± 13.60 **	2.89 ± 0.26 **	156.36 ± 25.89 **	24.60 ± 1.81 **
	5	2.17 ± 0.13 **^##^	322.16 ± 27.94 **	3.58 ± 0.25 ^##^	191.47 ± 18.69 *^#^	43.43 ± 2.39 *^##^
	10	1.60 ± 0.10 ^##^	385.12 ± 25.21 ^##^	4.31 ± 0.15 ^##^	220.12 ± 30.57 ^##^	53.19 ± 2.73 ^##^

Compared with the NC group * *p* < 0.05, ** *p* < 0.01. Compared with the MC group ^#^ *p* < 0.05, ^##^ *p* < 0.01.

**Table 2:** Effects of AHG on antioxidant activity in hearts from CY-treated mice (*n* = 8).

Group	Dose (mg/kg)	MDA (nmol/mg pro)	SOD (U/mg pro)	T-AOC (U/mg pro)	GSH-PX (U/mg pro)	CAT (U/mg pro)
NC		3.39 ± 0.23	256.93 ± 22.22	2.83 ± 0.09	83.01 ± 7.46	39.90 ± 3.01
MC		6.34 ± 0.13 **	187.77 ± 18.34 **	1.28 ± 0.1 **	57.11 ± 3.53 **	21.83 ± 2.56 **
LNT	1	3.64 ± 0.27 ^##^	251.19 ± 32.64 ^#^	2.83 ± 0.01 ^##^	80.06 ± 2.33 ^##^	40.28 ± 3.74 ^##^
AHG	1	5.74 ± 0.12 **^##^	208.02 ± 12.10 **	1.61 ± 0.08 **^##^	61.62 ± 7.57 *^#^	22.36 ± 1.56 **
	5	4.49 ± 0.30 **^##^	246.20 ± 22.57 ^#^	2.70 ± 0.08 ^##^	71.39 ± 4.57 *^#^	34.43 ± 1.34 **^##^
	10	3.44 ± 0.19 ^##^	256.86 ± 28.75 ^##^	3.28 ± 0.04 ^##^	83.39 ± 2.59 ^##^	40.66 ± 4.63 ^##^

Compared with the NC group * *p* < 0.05, ** *p* < 0.01. Compared with the MC group ^#^ *p* < 0.05, ^##^ *p* < 0.01.

**Table 3:** Effects of AHG on antioxidant activity in kidneys from CY-treated mice (*n* = 8).

Group	Dose (mg/kg)	MDA (nmol/mg pro)	SOD (U/mg pro)	T-AOC (U/mg pro)	GSH-PX (U/mg pro)	CAT (U/mg pro)
NC		2.42 ± 0.25	86.14 ± 0.57	3.32 ± 0.20	164.77 ± 12.47	51.75 ± 0.81
MC		4.38 ± 0.34 **	66.03 ± 1.04 **	1.72 ± 0.17 **	130.53 ± 3.38 **	36.50 ± 1.34 **
LNT	1	2.85 ± 0.26 *^##^	84.91 ± 1.44 *^##^	3.51 ± 0.22 ^##^	163.27 ± 8.48 ^##^	52.34 ± 2.25 ^##^
AHG	1	3.93 ± 0.25 **	77.59 ± 0.66 **^##^	2.40 ± 0.32 *^##^	136.90 ± 4.91 **^##^	41.95 ± 0.94 **^##^
	5	2.94 ± 0.25 *^##^	82.68 ± 1.94 **^##^	2.83 ± 0.21 ^##^	142.05 ± 7.90 **^##^	45.81 ± 2.57 **^##^
	10	2.31 ± 0.17 ^##^	89.75 ± 0.99 **^##^	3.47 ± 0.18 ^##^	158.40 ± 8.64 ^##^	53.00 ± 1.38 *^##^

Compared with the NC group * *p* < 0.05, ** *p* < 0.01. Compared with the MC group ^#^ *p* < 0.05, ^##^ *p* < 0.01.

## Supplementary Materials

Supplementary File 1
