# Supplementary figures and images for: Immunoenhancement Effects of Glycosaminoglycan from Apostichopus japonicus: In Vitro and In Cyclophosphamide-Induced Immunosuppressed Mice Studies
Source: Mar Drugs. 2017 Nov 7;15(11):347. doi: 10.3390/md15110347 (PMC5706037; doi:10.3390/md15110347)

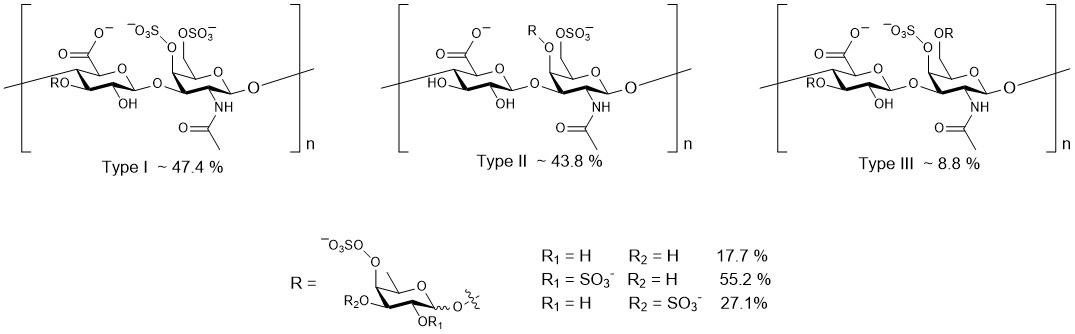

Supplement: Supplementary File 1 [file marinedrugs-15-00347-s001.zip › Supplementary Materials/Structure of AHG.jpg]
